# Do picture-based charts overestimate visual acuity? Comparison of Kay Pictures, Lea Symbols, HOTV and Keeler logMAR charts with Sloan letters in adults and children

**DOI:** 10.1371/journal.pone.0170839

**Published:** 2017-02-02

**Authors:** Nicola S. Anstice, Robert J. Jacobs, Samantha K. Simkin, Melissa Thomson, Benjamin Thompson, Andrew V. Collins

**Affiliations:** 1 School of Optometry and Vision Science, The University of Auckland, Auckland, New Zealand; 2 School of Optometry and Vision Science, University of Waterloo, Waterloo, Ontario, Canada; Durham University, UNITED KINGDOM

## Abstract

**Purpose:**

Children may be tested with a variety of visual acuity (VA) charts during their ophthalmic care and differences between charts can complicate the interpretation of VA measurements. This study compared VA measurements across four pediatric charts with Sloan letters and identified chart design features that contributed to inter-chart differences in VA.

**Methods:**

VA was determined for right eyes of 25 adults and 17 children (4–9 years of age) using Crowded Kay Pictures, Crowded linear Lea Symbols, Crowded Keeler logMAR, Crowded HOTV and Early Treatment of Diabetic Retinopathy Study (ETDRS) charts in focused and defocused (+1.00 DS optical blur) conditions. In a separate group of 25 adults, we compared the VA from individual Kay Picture optotypes with uncrowded Landolt C VA measurements.

**Results:**

Crowded Kay Pictures generated significantly better VA measurements than all other charts in both adults and children (p < 0.001; 0.15 to 0.30 logMAR). No significant differences were found between other charts in adult participants; children achieved significantly poorer VA measurements on the ETDRS chart compared with pediatric acuity tests. All Kay Pictures optotypes produced better VA (p < 0.001), varying from -0.38 ± 0.13 logMAR (apple) to -0.57 ± 0.10 logMAR (duck), than the reference Landolt C task (mean VA -0.19 ± 0.08 logMAR).

**Conclusion:**

Kay Pictures over-estimated VA in all participants. Variability between Kay Pictures optotypes suggests that shape cues aid in optotype determination. Other pediatric charts offer more comparable VA measures and should be used for children likely to progress to letter charts.

## Introduction

In pediatric eye care the measurement of visual acuity (VA) is central to the diagnosis of refractive error,[1] amblyopia[2] and pathology.[3] Nevertheless, factors such as the inability of many preschool children to name letters and individual differences in cognitive development complicate acuity measures.[4] Although a gold standard pediatric visual acuity system exists (the Electronic Visual Acuity (EVA) tester)[5] it is primarily used for clinical trials rather than mainstream clinical practice. It is unclear why the EVA tester has not been adopted more widely in general ophthalmic practice. However, its reduced utility for other clinical techniques, such as subjective refraction, coupled with increased cost, may diminish the appeal of the EVA system for many eye care providers.

International guidelines[6] have been employed in the development of many modern pediatric charts. However, differences between tests exist and understanding the influence of chart differences on VA measurements is important.[2, 7–9] For example, some picture optotype tests over-estimate VA results compared with letter tests,[10–13] due to optotype design[14] or because of the reduced number of pictures employed.[15] Such inter-chart differences have significant visual health implications. Vision screening referrals are commonly based on a pre-determined level of vision[16–18] and differences between chart designs may require chart-specific adjustment of this level. Additionally, children under ophthalmic care require accurate measurement of VA for the diagnosis of vision disorders and assessment of treatment outcomes. During childhood, vision may be measured using multiple charts and understanding if changes in VA are real change or an artefact of different chart designs is essential. Furthermore, the same child may be assessed by a variety of healthcare providers in different settings, each employing a different test. Therefore, eye care providers need a clear understanding of differences between charts to allow for accurate interpretation of VA measurements.

Building upon previous pediatric visual acuity chart comparisons [4, 9, 19] the primary objective of our first experiment was to compare within-subject VA measurements across four selected pediatric charts (Keeler logMAR, HOTV, Lea Symbols and Kay Pictures, all presented in crowded linear format) in adults and children under focused and defocused conditions. We also compared measurements from each pediatric chart to measures made using the gold standard ETDRS chart. Our hypothesis was that clinically significant differences in VA would be apparent across the pediatric charts and that only measures from a subset of charts would agree with the ETDRS chart. Furthermore, we hypothesized that pediatric charts would differ in the extent to which VA was influenced by defocus. We tested both children and adults because chart differences that were common to both groups would be unlikely to result from factors such as immature cognitive function or letter / picture naming ability.

The results from Experiment One indicated that logMAR VA thresholds using the Crowded Kay Pictures chart were significantly lower (better) than VA measures obtained with any of the other charts tested; VA measures on the other pediatric tests did not differ significantly from each other. The objective of Experiment Two was to further investigate this discrepancy by measuring VA thresholds for uncrowded individual Kay Pictures optotypes compared with an uncrowded Landolt C optotype with equivalent stroke width. The Landolt C target was chosen as the comparison optotype in this Experiment, as the ‘C’ optotypes are identical in all presentations, except for the position of the gap, and therefore should have equal discriminability. Our hypothesis was that VA for the Kay Picture optotypes was affected by factors other than stroke width, such as shape cues.[20]

## Materials and methods

### Study design

The research followed the tenets of the Declaration of Helsinki, the protocol was approved by the University of Auckland Human Participants Ethics Committee; all participants provided written informed consent (adults / parents) and assent (children).

The primary outcome for Experiment One was the direct comparison of VA measures between Kay Pictures, Lea Symbols, HOTV, Keeler logMAR and the ETDRS chart under focused conditions. Secondary outcomes included visual acuity comparisons under defocused conditions, testability of charts in children and the testing distance required to reach threshold acuity measures.

The primary outcome for Experiment Two was the comparison of VA measures for individual Kay Pictures optotypes. The secondary outcome was a comparison with single optotype Landolt C VA. Defocus was not used in Experiment Two.

### Experiment One

#### Participants

Twenty-five adults (mean (± SD) age 27 ± 8.8 years, age range 16–50 years of age) and 17 children (mean (± SD) age 6.7 ± 1.7 years, 4–9 years of age[21]) were recruited. *A priori* sample size calculations were performed to ensure that the number of participants was sufficient to obtain a power of 80% at the 5% level for detecting a difference of 0.15 logMAR between charts based on a standard deviation of 0.1 logMAR for differences in VA measures in children.[22–24] This sample size calculation indicated a minimum of six participants were required, however due to the potential for incomplete data sets, particularly for children, our recruitments target was initially 15 in each age group. As sample size calculations only estimate the required number of participants, and small differences in parameters can lead to large differences in sample size,[25] we recruited an additional 10 adult participants to ensure that the study had adequate power to detect a real difference if our estimate of standard deviation for adult VA measures on pediatric charts was incorrect.[26, 27]

Adult participants were recruited from the staff and students of the Faculty of Medical and Health Sciences, University of Auckland, while children were recruited through advertising within the University of Auckland and its Optometry Clinic. Participants were included if they had no vision problems, except the need to wear refractive correction, and VA of 0.10 logMAR or better in each eye. Visual acuity was verified using the ETDRS chart in adults and on the crowded Keeler logMAR chart in children, using versions of the chart not used for subsequent data collection. All participants who volunteered met the inclusion criteria and took part in the experiment. Prior to measurement of VA, manifest refractive error was measured using standard techniques (non-cycloplegic retinoscopy with +1.50 DS fogging lenses and subjective refraction) and corrected with trial lenses. Refraction was based on retinoscopy alone for the three youngest children in the study (two children 4 years of age and one 5 years of age) because subjective refraction was too cognitively demanding.

#### Apparatus

**Fig 1:**
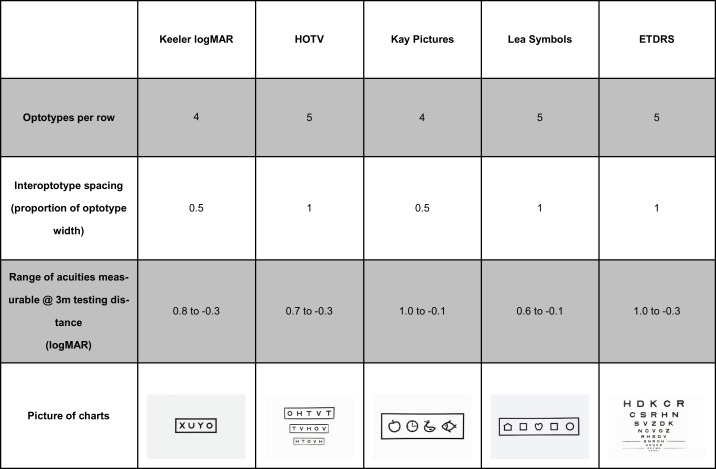
Key features of the acuity charts investigated.

Acuity measurements were made with four pediatric charts: multiple line crowded HOTV (HOTV Pediatric Eye Chart for the Wall using Massachusetts VA format, Good-lite Ltd), crowded Keeler logMAR (Keeler Ltd), crowded single line Lea symbols (Massachusetts Flip Chart, Good-lite Ltd) and the Crowded Kay Pictures chart (Kay Pictures Ltd). These measures were compared with the ETDRS chart (Original Series Chart 2, Precision Vision) (Fig 1).

The versions of the HOTV and Lea Symbols both employed the Massachusetts Visual Acuity Testing (MassVAT) format with crowding bars surrounding individual rows of optotypes. While single crowded optotypes are used in the gold standard Amblyopia Treatment Study testing protocol[2] single crowded versions of the Keeler logMAR and Kay pictures charts are not commercially available. Therefore, we chose to use crowded linear versions of each chart (a single line of letters or pictures surrounded by a solid black box) as this provided the most comparable version of the pediatric tests in Experiment One. While the HOTV chart had multiple lines, this was in a MassVAT presentation,[4] which is recognized as an appropriate format for testing children’s acuity.[28]

#### Procedure

Right eye VA was measured with manifest refractive correction in place (focused condition) and with additional optical defocus (+1.00 DS lens to explore Chart-Defocus interactions) in a single session. The left eye was covered by an occluder in adults and an eye patch in children. Prior to data collection, children matched or named specified optotypes on each chart at near (40–50 cm) under binocular conditions. This allowed assessment of whether the child was able to perform the acuity measurement task required by each chart.[4] Children unable to perform an acuity measurement on any chart(s) were not excluded from the study but an inability to provide measurements was noted. Pretesting was not conducted for adults.

Charts were presented in a random order which was different for each participant, with VA measurements in focused conditions, the primary outcome always preceding measurements in defocused conditions. Prior to data collection, to ensure threshold measurements were obtained for each chart and to avoid truncation of acuity measures, the testing distance was increased in log unit steps from a starting distance of 3 m until the smallest line was no longer recognized. To avoid participants memorizing the order of optotypes, test distance calibration was conducted with a version of the chart not used for data collection which had the same design but a different optotype order. The new testing distance was used as the distance for focused and defocused conditions and logMAR VA results were adjusted accordingly. Participants’ ability to memorize optotype order was minimized as measurements in the focused condition were collected on all five charts, prior to any measurements in the defocused condition.

Starting with the largest line, participants identified the first three optotypes on each line of the chart until an incorrect response was given. After this error, participants were then asked to read the remaining two optotypes on the line and then all successive optotypes on the chart. Participants were encouraged to guess the optotype if unsure and termination of testing occurred when the participant declined to identify any smaller optotypes. We employed an optotype-by-optotype scoring system giving credit for all correctly identified optotypes.

Data collection took approximately 45 minutes per participant for adults and 60 minutes per participant for children with regular breaks to avoid fatigue. Children received a sticker chart at the commencement of acuity testing and received a sticker after each completed VA measurement. This allowed them to assess their progress during data collection and provided a small incentive for completing the tasks.

#### Data analysis

After correcting for the actual viewing distance used, VA was scored based upon the last line completed correctly (all of the first three optotypes read correctly), plus either -0.02 log units (charts with five optotypes per line) or -0.025 log units (charts with four optotypes per line) for each additional correct response. Standard optotype-by-optotype scoring was used, with no adjustment for differences in guessing rates between charts,[15] as this best reflects actual clinical VA measures of tests. The mean VA measures for each chart were evaluated with an omnibus two-way repeated measures Analysis of Variance (ANOVA) using the within-subject factors of Chart and Focus (focused or defocused) and the between-subjects factor of Group (adults versus children). Where the within subjects or the between subjects main factors were found to be statistically significant, Bonferroni corrected (adjusted p < 0.005) post-hoc comparisons were used to further explore differences in VA measures. Pre-specified Chart-Focus and Chart-Group (adults versus children) interactions were also explored within the model to analyze whether defocus or age-group influenced VA measures on any of the charts investigated. VA measures between charts were compared following Bland Altman guidelines.[29] All analyses were conducted in SPSS v22 (IBM, New York, USA). As previous studies have shown mean VA measures on the ETDRS chart vary with age in children <13 years,[30] the relationship between age and measured VA, as well as age and chart testing distance, was explored for children using correlation analysis.

### Experiment Two

#### Subjects

A different group of 25 adults, mean 24.7 ± 6.2 years of age, with VA of 0.00 logMAR or better in each eye in their habitual correction, as measured on a Medmont AT20R computerized acuity tester (Medmont Pty Ltd, Australia), and no self-reported history of ocular pathology participated. All participants who volunteered were recruited. Data were collected for the right eyes of all participants, with refractive correction in place, if required. No formal sample size calculations were performed as differences between, and variability within, Kay Pictures optotypes were unknown. Convenience sampling techniques were used for recruitment purposes and our final sample was larger than other similar studies.[14]

#### Procedure

A high contrast facsimile of each Kay Picture was constructed at a fixed size equivalent to the 0.00 logMAR line so that the stroke width of the optotype subtended a visual angle of 1 minute of arc at 3 m. Individual uncrowded optotypes were then presented in a random order at viewing distances varied in logarithmic steps (e.g. 3.8 m, 4.8 m, 6.0 m, 7.5 m, 9.0 m etc.) until the participant’s correct identification rate approached the guessing rate (< 12.5%) of the task. Each optotype was presented five times at each viewing distance. Random presentation was determined prior to presentation of optotypes using a random number generator and a simple (unrestricted) randomization approach.[31] The threshold viewing distance for each Kay Picture optotype was found by fitting a polynomial function to the number of correct responses as a function of viewing distance. Taking into account the one in eight chance of correctly guessing each optotype, threshold was defined as the viewing distance corresponding to at least 56% correct identification. After obtaining the threshold viewing distance for each Kay Pictures optotype for each participant, the resultant Snellen fractions (test distance (3 m) / threshold viewing distance) were converted to logMAR equivalents for comparison with a Landolt C test.[6] The Landolt C was chosen for Experiment Two as it represents a more consistent task than both the EVA and ETDRS systems as Sloan letters have unequal legibility.[32, 33]

The uncrowded Landolt C target was presented on a Medmont AT20-R chart at a viewing distance of 6 m using a four alternate forced choice paradigm (orientations up, down, left and right). Each optotype size was presented five times and VA was scored using the optotype-by-optotype method giving credit for all optotypes read correctly. As we were not comparing the VA measures obtained with different orientations of the Landolt C task, but simply as a reference test for comparison with the individual Kay Pictures, the guessing rate was not taken into consideration when scoring this test. Termination of testing occurred when participants identified less than three optotypes correctly at a given acuity level. Comparison of VA measures with the Landolt C with each of the Kay Pictures optotypes was performed with a repeated measures ANOVA with post-hoc Bonferroni correction (adjusted p value 0.002).

## Results

### Experiment One

#### Testability of charts in children 4–9 years of age

Adult participants completed all measures in Experiment One; testability with child participants is shown in Table 1. All children completed binocular pretesting for the pediatric acuity charts however two four-year-old children were unable to perform pre-testing with the ETDRS chart, and VA measurement on the ETDRS was not attempted. In general, the testability under defocused conditions was poorer (Table 1). Three children 4–5 years of age, one child seven years of age and one child 9 years of age refused to attempt VA measurements on one or more charts in the defocused condition.

**Table 1:** Testability of charts in a group of 17 children 4–9 years of age.

Chart	Number (percentage) of children able to complete measurements in focused conditions	Number (percentage) of children able to complete measurements in defocused conditions
**ETDRS**	15 (88%)	12 (71%)
**Keeler logMAR**	17 (100%)	14 (82%)
**HOTV**	17 (100%)	14 (82%)
**Lea Symbols**	17 (100%)	15 (88%)
**Kay Pictures**	17 (100%)	13 (76%)

#### Testing distances required to achieve threshold VA

**Fig 2:**
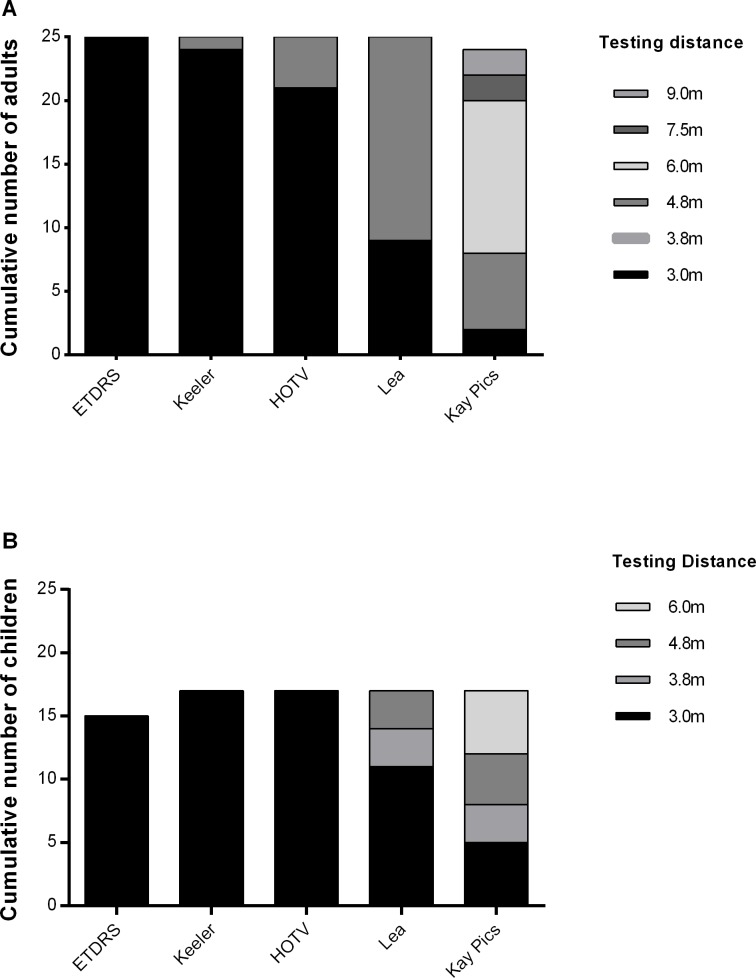
Number of participants able to reach threshold acuity measurements on each of the pediatric tests for the different test distances shown. Only the ETDRS chart allowed for threshold measurements at the standard (3 m) testing distance for all participants. The number of children able to provide results on the ETDRS chart was less.

All participants obtained threshold acuity measurements at the standard 3 m testing distance on the ETDRS chart. Other charts required distances greater than the manufacturers’ recommended testing distances to avoid truncation effects (Fig 2). For the Kay Pictures chart, approximately 1/3 of children and 2/3 of adult participants required an extended testing distance of at least 0.3 log steps (double the distance) to obtain VA thresholds.

#### Comparison of VA between charts in both adults and children

**Fig 3:**
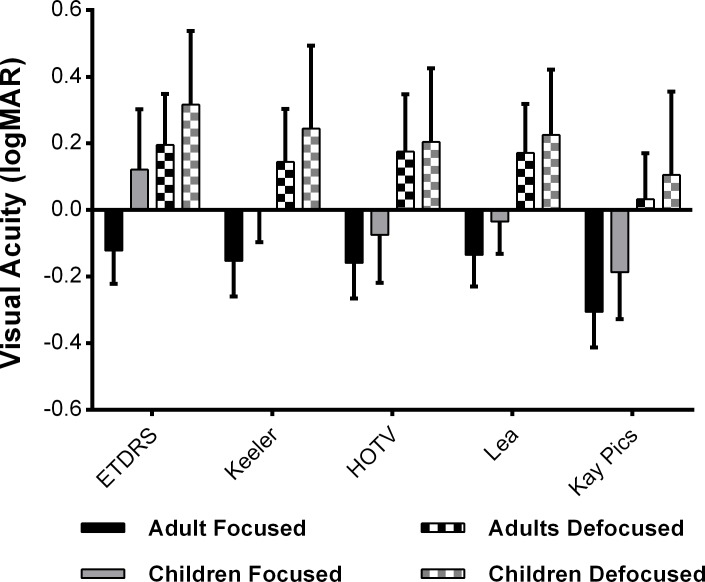
Mean (± standard deviation) of VA measurements for adults (white) and children (light grey) in focused conditions and defocused (adults black and children dark grey) conditions.

The mean (± standard deviation) VA for each chart under focused and defocused conditions is shown in Fig 3. Repeated measures ANOVA showed significant main effects of Chart (p < 0.0001), Focus (p < 0.0001) and Group (p = 0.008). VA thresholds measured with the Crowded Kay Pictures chart were significantly lower (produced better VA) than all other tests (p < 0.001). No other significant differences were found in post-hoc analysis of adult data. In children, VA measurements with the ETDRS chart were significantly worse than the pediatric charts investigated. Optical defocus increased VA thresholds (poorer performance) by 2.5–3 lines, however it affected all charts equally (Chart-Defocus interaction p = 0.106).

**Fig 4:**
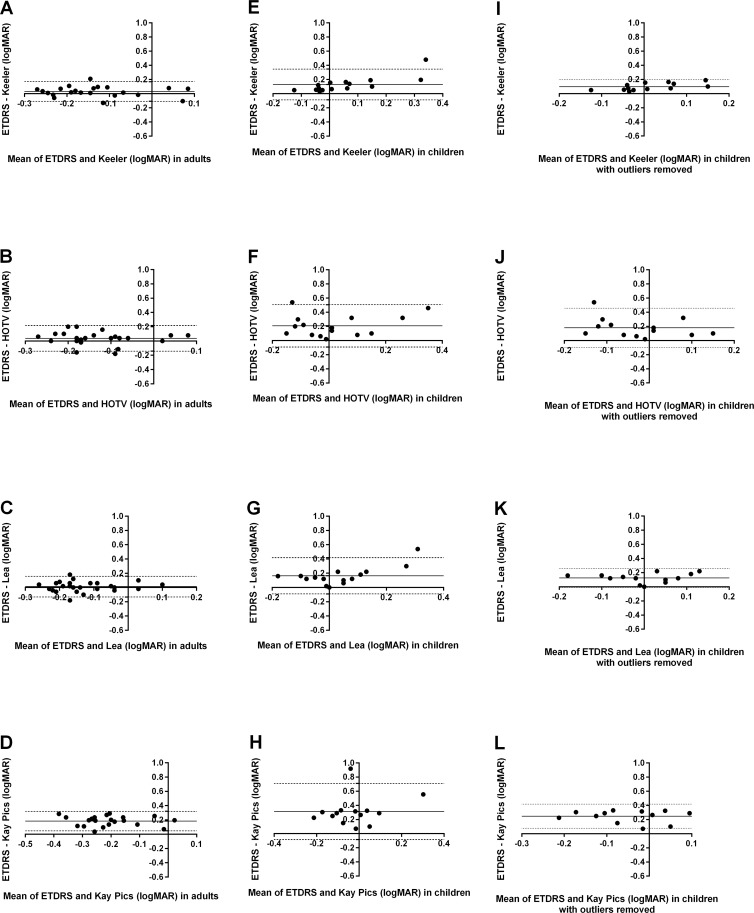
Bland Altman comparisons of visual acuity measurements between ETDRS and pediatric charts in adults (A-D) and children (E-H and I-L with two outliers removed). A positive bias indicates the second chart gave better acuities.

In adults, VA measures on the Keeler logMAR, HOTV and Lea symbols charts (Fig 4A–4C) were equivalent to VA measures on the ETDRS chart with a systematic bias close to 0 (solid line). Bland Altman analysis revealed wider 95% limits of agreement (dashed lines) in children (Fig 4E–4H), with the Kay Pictures chart showing the greatest variability. Two children had poor measures of VA recorded with ETDRS test (0.42 logMAR and 0.58 logMAR on ETDRS). While these two data points were not statistical outliers (Rout analysis with Q = 0.1%), clinically these two children had significantly better VA measures on age-appropriate tests. Bland Altman analysis was repeated for the pediatric data with these two data points removed (Fig 4I–4L). There was no proportional bias in measured VA between the ETDRS chart and any of the pediatric tests charts. In children, there appeared to be significant proportional bias found between VA measures on the ETDRS chart and the crowded Keeler logMAR (p = 0.004) and Lea Symbols (p = 0.006). However, this relationship was driven by the two children with poor acuity on the ETDRS chart and once these data were removed no proportional bias was observed.

#### Effect of age on VA testing in children

There was a negative correlation between age and measured VA on the ETDRS (r = -0.59, p = 0.019), Keeler logMAR (r = -0.63, p = 0.006) and Lea symbols charts (r = -0.61, p = 0.009) indicating logMAR VA improved with age, but no significant correlation between age and VA on the HOTV (r = -0.48, p = 0.053) and the Kay Pictures (r = -0.028, p = 0.914). The standard testing distance (3 m) was used for all letter charts so there was no association between age and testing distance for the ETDRS, Keeler logMAR or HOTV charts. There was a significant positive correlation between age and the testing distance used for the Lea Symbols chart (r = 0.57, p = 0.016) suggesting older children required a longer working distance to avoid truncation effects, but no relationship for the Kay Pictures chart (r = 0.192, p = 0.461).

### Experiment Two

#### Discriminability of Kay Pictures optotypes compared with Landolt C

**Fig 5:**
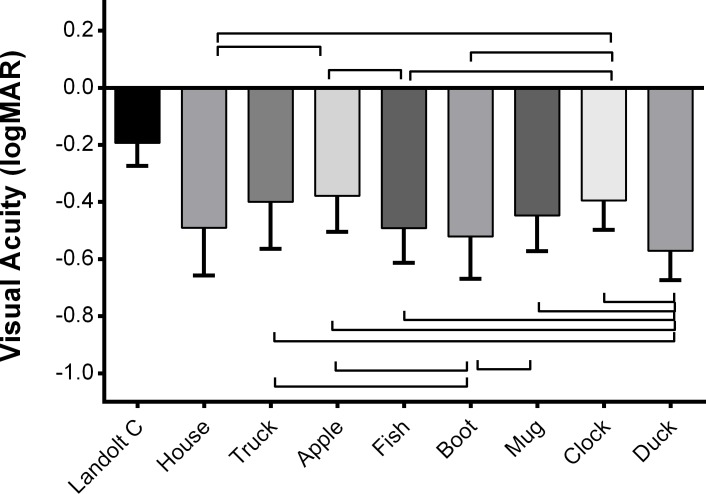
Visual acuities provided by individual Kay Pictures optotypes compared with a Landolt C target. All Kay Pictures were more discriminable than the Landolt C. Significant differences (p < 0.002) between individual Kay Pictures are indicated on the figure.

All Kay Pictures optotypes provided better VA thresholds than the reference Landolt C (p < 0.0001; Fig 5). Variability in threshold results between Kay Pictures optotypes, from -0.38 ± 0.13 logMAR for the apple to -0.57 ± 0.10 logMAR for the duck, meant some optotypes needed to be viewed from greater distances than others to reach threshold size.

## Discussion

### The crowded Kay Pictures chart overestimates VA

This study compared pediatric acuity tests more commonly used in the United States (Lea Symbols and HOTV) with similar charts commonly used in British practice (Kay Pictures and Keeler logMAR). This comparison may be useful for clinicians who wish to assess their treatment results against published studies which have used an alternative acuity chart, or for researchers who wish to compare studies, particularly those that have been conducted in trans-Atlantic countries. Our results indicate that the Crowded Kay Pictures overestimate VA compared with the ETDRS chart and other pediatric acuity tests investigated. To the best of our knowledge our study is the first to compare the Crowded Kay Pictures and Lea Symbols charts, providing valuable information on the relative performance of these two picture optotype tests. We found a greater VA difference between the Kay Pictures and letter charts than other authors[11, 20, 21, 34] which may be because (i) we corrected manifest refractive error prior to data collection and (ii) we increased the testing distance of charts to avoid truncation effects. The effect of defocus did not vary significantly across the different charts we tested, suggesting that the Kay Pictures chart responds to defocus in the same way as other tests. However, as the measured VA in focused conditions was significantly better on the Kay Pictures chart, acuity was worse than 0.2 logMAR[20] in only two adults and five children when +1.00 DS of optical defocus was introduced. This is in agreement with other studies which have found that significant amounts of optical defocus have been needed to worsen VA measured with the Kay Pictures test.[20]

**Fig 6:**
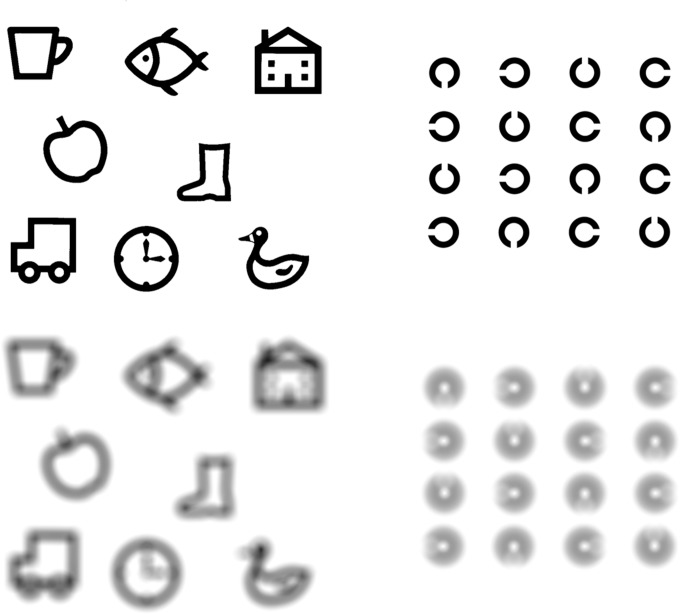
Demonstration of focused and defocused Kay Pictures optotypes (upper and lower left, respectively) compared with a reference Landolt C optotype (upper and lower right). The stroke width, nominal acuity and amount of defocus are equal in the Kay Pictures and Landolt C targets; however, Kay Picture optotypes, because of their construction principles, are at least twice the size of the Landolt C. Images were constructed in Adobe Photoshop CS6 v13 using the Lens Blur filter.

Experiment Two confirmed the previously reported dissimilarity of individual Kay optotypes and suggests that additional information aids optotype recognition resulting in VA measurements better than would be predicted based upon stroke width alone.[14] Due to the dissimilarity of individual Kay optotypes, a simple conversion from Kay Pictures to other charts is not possible as the measured VA on the Kay Pictures test will vary based on the four optotypes displayed at each size. While VA measures under defocused conditions were not undertaken in Experiment Two, Fig 6 demonstrates that in the presence of simulated lens defocus (Lens Blur filter in Adobe Photoshop CS6 v13 (Adobe Systems Inc., USA)), identification of Kay optotypes is potentially aided by shape cues which are not available with the reference Landolt C task. Although Experiment Two investigated optotype discriminability only in adult participants, data from Experiment One suggests that children also use cues other than stroke width to identify the target Kay Pictures. While it would have been ideal to collect individual optotype discriminability data in a cohort of children, Experiment Two was not repeated in children as a descending method of limits psychophysical testing technique was employed which resulted in an increased time required for data collection.

Other studies have reported VA measures on the Kay Pictures chart that are comparable to those that might be expected from other pediatric acuity tests. Saul and Taylor (2015) found that the mean Crowded Kay Pictures VA for visually normal 3–5 year old children (n = 110) was 0.108 ± 0.062 logMAR, with all children having VA poorer than -0.05 logMAR avoiding a truncation effect of the test.[35] Likewise, Cross et al. (2015) found a median Crowded Kay Pictures VA of 0.05 logMAR (Interquartile Range 0.00–0.10 logMAR) in 3–4 year old children (n = 733) with no significant refractive errors.[36] Kay pictures appeared to identify large refractive errors but failed to detect some children with borderline ametropia. Both studies were large population based studies and are therefore not directly comparable to our work but show that pediatric VA measures can vary depending on cohort characteristics, for example age of participants, refractive error and presence of amblyopia. The sample of children we recruited comprised children with no known ocular conditions, other than refractive error, and VA measures were performed with optimal refractive correction in place. This may explain the differences in VA measures seen between other published work and our data.

In our cohort, VA measurements with the HOTV, Keeler logMAR and Lea Symbols tests were equivalent.[37] [38] However, Mercer et al. (2013) found that the Lea Symbols over-estimated VA compared with Patti Pics and Sloan letters[10] but this was using a line-by-line scoring method, whereas we used optotype-by-optotype scoring which may explain the difference between results.[22] Lea symbols were designed so that VA measured with each individual optotype was highly correlated with measured VA for the whole test [7] and recent evidence has confirmed statistically equal pairwise similarity between optotypes.[14] This may help explain the similarity between measured VA with the ETDRS and Lea symbols charts in our sample. In our child participants, the mean VA measured on the ETDRS chart was similar to other published data,[39, 40] however, the testability of children for the ETDRS chart was less than for other charts.[41] [42] Optical defocus reduced the likelihood of obtaining VA measures in children for all charts investigated.

### Use of adult participants

Like other published work[10] we assessed which of four pediatric VA tests adhered most closely to the international adult gold standard—the ETDRS chart. Although the four pediatric charts examined are primarily designed for use in preschool children, the experiments were carried out on adults, as well as some older children, an approach undertaken by other authors [14, 20, 43, 44]. VA is not the same in adults and children, with children’s acuity often 1–2 lines worse than adults[24] due to the more pronounced effect of crowding in childhood.[21] However, the relationship between VA measures obtained across pediatric charts is similar in adults and children,[10] suggesting that data obtained for adults may be applicable to preschool children for whom the charts are designed.[44]

While the pediatric acuity tests investigated are primarily designed to be used with young children (typically 3–5 year olds), adult data is important as it explores the fundamental differences between tests without potential confounding factors such as cognitive development and attention span.[14] While interpreting clinical results for children may be difficult based on data provided by adults, our adult data is consistent with the pediatric results in our study which suggests the acuity differences seen with Kay Pictures occur in all both children and adults. Although some of the children in Experiment One were older than children traditionally tested with pediatric charts one of our outcome measures was the comparison of VA measures on pediatric charts with the ETDRS chart. The testability of the ETDRS chart in preschool children is generally poor with < 47% of children under 5.5 years of age able to complete testing in a recent Australian study.[45] We therefore included older children (up to nine years of age) to allow for better comparison with ETDRS data. Our data now needs to be confirmed by testing a larger sample of children, within an appropriate age range, in a pediatric setting. However, it is unlikely that all children will be able to provide full data sets and a large sample size may be required to achieve sufficient statistical power.

### Limitations of the study

Our study was small, however *a priori* power calculations were performed to determine sufficient sample sizes to detect a clinically significant difference.[23] Nevertheless, all participants were visually normal and our data do not necessarily provide information about the relative performance of charts in children with amblyopia or other forms of visual impairment. Likewise, cycloplegia has been used in some studies[20, 46] to control the amount of defocus presented. We used positive lenses to stimulate refractive error but acknowledge, particularly in pediatric patients, that this may have left some latent hyperopia uncorrected. However, this should have affected VA equally across charts as measurements were completed in a single session for all participants.

In order to measure VA accurately and avoid truncation effects in Experiment One, we utilized a variable working distance for charts based on individual participants’ threshold VA with each test. To establish a true threshold acuity, it was important to use a test distance that was sufficient to ensure an accurate measurement of VA was established and extended testing distances in Experiment One were required for both adult and child participants. Establishing a precise measure of VA by avoiding truncation effects of the test is important in children, particularly when it is likely that the child will progress to letter acuity charts in the future. Therefore, ensuring we used a testing distance that allowed for a precise assessment of VA in both adults and children was required for the correct interpretation of data in Experiment One. This alters the accommodative demand of the test with 0.33 D of accommodation required at a 3 m testing distance but only 0.11 D required at the greatest testing distance used (9 m). Others authors[20, 21] have used a standard 6 m testing distance of all charts to ensure that accommodative demand was equivalent for all participants. However, this would have induced a truncation effect for 4 of our adults on the Kay Pictures test.

The use of crowded acuity charts is recommended as soon as feasible in pediatric eye care due to their increased sensitivity for detecting vision problems.[20] However, the multiple line ETDRS is not recommended for use in preschool children because of low testability and was the most difficult task for younger children to perform due to the unfamiliarity of letters and increased number of possible alternatives.[40, 47] Nevertheless, one of our secondary outcomes was comparison of VA measured on pediatric charts with the adult gold standard ETDRS chart. This information is valuable as it provides data on the expected change in VA when children are moved from pediatric picture or letter charts to standard adult charts during their ophthalmic care. Our data from Experiment One suggests that clinicians can expect a reduction in measured VA of 1–2 lines of letters if moving from HOTV, Keeler logMAR or Lea symbols to the ETDRS chart or approximately three lines of letters if moving from the Kay Pictures chart, in agreement with Shah et al., 2012.[11]

Data collection took approximately 60 minutes for the children who participated in Experiment One. While the authors involved in data collection had extensive experience working with preschool children,[48–50] fatigue and loss of concentration towards the end of the testing protocol could have influenced VA measurements. Chart presentation was randomized to prevent the effects of fatigue from affecting the results of Experiment One but it is possible that some children became tired during testing and this may have affected the reliability of some results. VA measurement in the focused condition always preceded measurements in the defocused condition to maximize cooperation with children. However, it is possible that some participants memorized optotype order; this was minimized by testing all five charts in the focused condition before defocused measurements. For all charts, participants were asked to read the first three optotypes on each line commencing with the largest letter or picture. Therefore, participants read approximately 150 optotypes across all charts prior to measurements in the defocused condition. To further minimize chart memorization, the order of chart presentation was the same in the focused and defocused conditions, so charts were not sequentially presented in the focused and defocused conditions.

## Conclusion

In summary, we found the Crowded Kay Pictures chart over-estimated VA which may be due to the overall shape cues available within each picture as has been reported in other studies[20]. The other pediatric charts investigated produced consistent measures of VA and, based on the four pediatric charts examined, Lea Symbols are more appropriate for children who are likely to progress to letter-based charts. Redesign of the Kay Pictures so the pictures are more equally discriminable may be possible. However, in its current format, VA results from the Crowded Kay Pictures chart should be interpreted with caution when compared to other tests.
